# Use of a temporary immersion bioreactor system for the sustainable production of thapsigargin in shoot cultures of *Thapsia garganica*

**DOI:** 10.1186/s13007-018-0346-z

**Published:** 2018-09-08

**Authors:** Carmen Quiñonero López, Patricia Corral, Bénédicte Lorrain-Lorrette, Karen Martinez-Swatson, Franck Michoux, Henrik Toft Simonsen

**Affiliations:** 1 Department of Biotechnology and Biomedicine, Faculty of Bioengineering, Technical University of Denmark, Lyngby, Denmark; 2 Department of Plant and Environmental Sciences, Faculty of Science, University of Copenhagen, Copenhagen, Denmark; 3 Evonik Advanced Botanicals SAS, Parçay Meslay, France; 4 Natural History Museum of Denmark, University of Copenhagen, Copenhagen, Denmark

**Keywords:** *Thapsia garganica*, Thapsigargin, Nortrilobolide, In vitro shoot cultures, Temporary immersion bioreactor, Sesquiterpenes, *TgTPS2* and *TgCYP76AE2*

## Abstract

**Background:**

Thapsigargin and nortrilobolide are sesquiterpene lactones found in the Mediterranean plant *Thapsia garganica* L. Thapsigargin is a potent inhibitor of the sarco/endoplasmic reticulum calcium ATPase pump, inducing apoptosis in mammalian cells. This mechanism has been used to develop a thapsigargin-based cancer drug first by GenSpera and later Inspyr Therapeutics (Westlake Village, California). However, a stable production of thapsigargin is not established.

**Results:**

In vitro regeneration from leaf explants, shoot multiplication and rooting of *T. garganica* was obtained along with the production of thapsigargins in temporary immersion bioreactors (TIBs). Thapsigargin production was enhanced using reduced nutrient supply in combination with methyl jasmonate elicitation treatments. Shoots grown in vitro were able to produce 0.34% and 2.1% dry weight of thapsigargin and nortrilobolide, respectively, while leaves and stems of wild *T. garganica* plants contain only between 0.1 and 0.5% of thapsigargin and below detectable levels of nortrilobolide. In addition, a real-time reverse transcription PCR (qRT-PCR) study was performed to study the regulatory role of the biosynthetic genes HMG-CoA reductase (*HMGR*), farnesyl diphosphate synthase (*FPPS*), epikunzeaol synthase (*TgTPS2*) and the cytochrome P450 (*TgCYP76AE2*) of stem, leaf and callus tissues. Nadi staining showed that the thapsigargins are located in secretory ducts within these tissues.

**Conclusions:**

Shoot regeneration, rooting and biomass growth from leaf explants of *T. garganica* were achieved, together with a high yield in vitro production of thapsigargin in TIBs.

**Electronic supplementary material:**

The online version of this article (10.1186/s13007-018-0346-z) contains supplementary material, which is available to authorized users.

## Background

Thapsigargins are sesquiterpene lactones that are widespread within the Mediterranean genus *Thapsia* (Apiaceae) [1]. Among this family of natural compounds, thapsigargin and nortrilobolide are present in the species *Thapsia garganica* and *Thapsia gymnesica* [2]. Thapsigargins are potent inhibitors of the mammalian sarco/endoplasmic reticulum Ca^2+^-ATPase (SERCA) [3]. When the molecules bind to the transmembrane of mammalian cells, SERCA is prevented from maintaining a low concentration of Ca^2+^ in the cytosol and a high Ca^2+^ concentration in the reticulum leading to apoptosis [3]. A subnanomolar affinity for SERCA has made thapsigargin the most intensely studied molecule of the family [4].

Due to its ability to kill mammalian cells, thapsigargin has been used as a therapeutic target to induce apoptosis in cancer cells with a low rate of cell proliferation, thus with resistance to standard anti-proliferative chemotherapy. To target thapsigargin to the cancer cells, thapsigargin is fused with a masking peptide, which inhibits its biological activity until proteolytic cleavage at the tumor site [5]. Based on this technology, InSpyr Therapeutics (Westlake Village, California, previously GenSpera) has patented a thapsigargin-based prodrug named Mipsagargin (G-202). Mipsagargin has been studied in a Phase 2 clinical trial in patients with hepatocellular carcinoma [6], but the current status of the trials is unclear.

Marketing of a thapsigargin-based drug will increase the demand for this molecule. Until now, all of the commercially available thapsigargin is obtained from fruits and roots of wild *T. garganica* [4]. Traditional field cultivation techniques are very difficult and not economically viable for *T. garganica,* as with many species of Apiaceae, since the plants are difficult to germinate from seeds [7]. ThapsIbiza (Islas Baleares, Spain) is the only company that has started a small production of *T. garganica* plants. On the other hand, the total synthesis of thapsigargin was described in 2007. The approach allowed the total synthesis of thapsigargin in 42 steps from (s)-carvone with an overall yield of 0.6% [8], and recent developments have cut this to 12 steps with a 5.8% yield [9] and 11 steps with an overall yield of 0.137% [10]. Despite the successful synthesis of thapsigargin, the many steps for obtaining the core of the structure and the cost of the starting material make this approach commercially challenging. The biosynthesis of thapsigargins is not established and thus heterologous production in e.g. yeast or mosses is not yet possible [4, 11, 12].

Plant tissue culture technologies can provide an alternative production platform of thapsigargins and ensure a stable supply of thapsigargin at an industrial scale, using the temporary immersion bioreactors (TIB) developed at Alkion Biopharma (now part of Evonik Botanicals) [13] and is a prerequisite for plant transformation. TIBs have many advantages regarding other conventional in vitro plant propagation techniques. TIBs reduce manual labor, the risk of contamination and therefore the cost of the process. TIBs also avoid asphyxiation and tissue vitrification by exposing the plants to the liquid medium with periodic immersions, which ensures a complete renewal of the atmosphere. Plant growth and development can be controlled by modifying the frequency and duration of the immersion.

Only a few reports have been published on in vitro cultures of *T. garganica* since the first in 1993 [14]. A cell culture was established in which somatic embryos were spontaneously formed and followed by the development of roots and shoots [14]. However, only nortrilobolide and trilobolid were found in the tissue cultures obtained in this study [14]. Later, Makunga et al. [15] published protocols for the micropropagation of *T. garganica* and acclimation of ex vitro plants. This was followed by the in vitro regeneration of *T. garganica* from leaf explants via direct organogenesis [16] and with improved rooting and hyperhydricity in regenerating tissues [17]. Nonetheless, thapsigargin was not detected or measured in these studies. Thus, to the best of our knowledge thapsigargin production has not been established in in vitro cultures prior to this work. The main objective of this study was to establish and evaluate an efficient production platform of thapsigargin based on in vitro tissue culture techniques. We established in vitro shoot cultures that can be cultivated at an industrial scale in TIBs. The culture’s ability to produce thapsigargin and nortrilobolide was quantified by analytical chemistry techniques (HPLC and UPLC-MS) along with the gene expression (qRT-PCR) of the biosynthetic genes: *HMGR*, *FPPS*, *TgTPS2* and *TgCYP76AE2*. Analysis with Nadi staining was also performed in the tissue cultures.

## Results

### In vitro culture establishment

**Fig. 1:**
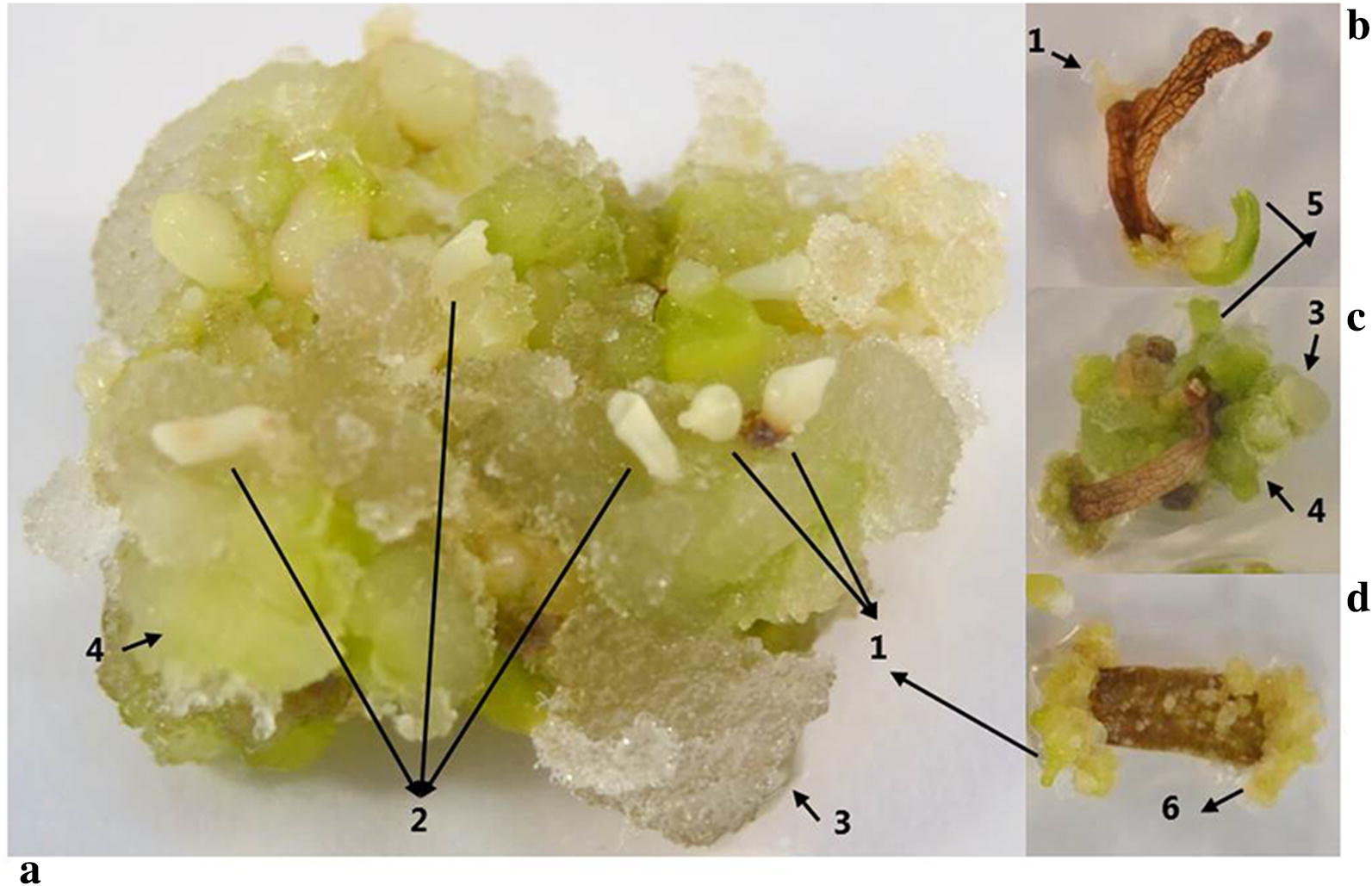
*T. garganica* leaf explants after 12 weeks in MS with different plant growth regulators (plant growth regulators). **a** TDZ (0.1 mg/L) + 2,4-D (0.1 mg/L), 16 h light photoperiod yielding calli type I (3) and III (4), and presence of embryos in different stages (heart (1) and torpedo (2) stages) and shoot buds (1). **b** BAP (0.1 mg/L) + 2,4-D (0.1 mg/L), 16 h light photoperiod yielding the presence of embryos (1) and a shoot (5). **c** TDZ (1 mg/L) + 2,4-D (0.1 mg/L), 16 h light photoperiod yielding calli type I (3) and III (4), and presence the of shoot bud (5). **d** TDZ (0.5 mg/L) + 2,4-D (0.1 mg/L), darkness conditions yielding calli type I and II (6) and the presence of embryos (1). plant growth regulator

Among the different treatments that were applied to leaf explants, only treatments combining auxin (2,4-Dichlorophenoxyacetic acid (2,4-D)) along with cytokinins (1-phenyl-3-(1,2,3-thiadiazol-5-yl) urea (TDZ) or N-(Phenylmethyl)-7H-purin-6-amine (BAP)) responded positively. From these, somatic embryogenesis and calli development were quantified (Table 1). Direct and indirect shoot regeneration was observed in some of the cultures (Fig. 1a), but no significant statistical differences between different concentrations of combined treatments were noted.

**Table 1:** The effect of different plant growth regulators on the induction of callus and somatic embryos from leaf explants of *T. garganica*

Treatment, plant growth regulators (mg/L)			Explant forming callus (%)		Type of callus		Explant forming embryos (%)		Number of embryos	
TDZ	BAP	2,4-D	Light	Dark	Light	Dark	Light	Dark	Light	Dark
0.1			16.7 ± 10^cd^	0^d^	IV (+)	–	0^f^	0^f^	0^e^	0^e^
0.5			10 ± 10 ^cd^	0^d^	IV (+)	–	0^f^	0^f^	0^e^	0^e^
1			43.3 ± 10^bc^	0^d^	IV (+)	–	0^f^	0^f^	0^e^	0^e^
0.1		0.1	96,7 ± 3.3^a^	96.7 ± 3.3^a^	I, II (+++)	I, III (++)	56.7 ± 3.3^a–d^	83.3 ± 3.3^a^	17.5 ± 5.5^a–d^	31.0 ± 5.0^a^
0.5		0.1	100^a^	100^a^	I, II (+++)	I, III(+++)	43.3 ± 10^b–e^	66.7 ± 6.7^a–c^	8.0 ± 3.0^c–e^	17.5 ± 3.5^a–d^
1		0.1	96.7 ± 3.3^a^	100^a^	I, II (++)	I, III (+++)	26.7^d–f^	60.0 ± 13.3^a–c^	4.5 ± 0.5^de^	11.5 ± 2.5^b–e^
	0.1		0^d^	0^d^	–	–	0^f^	0^f^	0^e^	0^e^
	0.5		0^d^	0^d^	–	–	0^f^	0^f^	0^e^	0^e^
	1		0^d^	0^d^	–	–	0^f^	0^f^	0^e^	0^e^
	0.1	0.1	73.3 ± 13.3^ab^	83.3 ± 10^ab^	I, II (+)	I, III (+)	40.0^c–e^	76.7 ± 3.3^a^	12.5 ± 3.5^b−e^	28.5 ± 5.5^a^
	0.5	0.1	93.3 ± 6.7^a^	96.7 ± 3.3^a^	I, II (++)	I, III (++)	13.30^ef^	73.3^ab^	4.5 ± 1.5^de^	20.0 ± 1.0^a−c^
	1	0.1	63.3 ± 23.3^ab^	86.7 ± 6.7^a^	I, II (++)	I, III (++)	16.7 ± 16.7^ef^	80.0 ± 6.7^a^	4.0 ± 4.0^de^	24.5 ± 2.5^ab^
		0.1	0^d^	0^d^	–	–	0^f^	0^f^	0^e^	0^e^

Type I: friable, soft white calli; type II: nodular green organogenetic and compact calli; type III: creamy, yellow nodular calli; type IV: brown dead calli. Furthermore, the amount of calli was noted from low to high (−, +, ++, +++)

Values represent mean ± SE. Different letters within a column indicate significant differences revealed after an ANOVA analysis followed by a Tukey’s multiple comparison test (*p* ≤ 0.05). Percentage values were arcsine transformed prior to analysis. n = 30 per treatment

The number of embryos produced per leaf explant varied with the concentration of cytokinins applied. Treatments with the lowest hormone concentrations, 2,4-D (0.1 mg/L) + TDZ or BAP (0.1 mg/L) yielded the highest number of somatic embryogenesis (Table 1). However, low concentrations of auxin 2,4-D or cytokinins (TDZ or BAP) alone, did not induce any organogenic response (Table 1). The photoperiod also influenced somatic embryogenesis. Leaf explants in total darkness seemed to produce more somatic embryos than explants grown in light. The continuous dark treatment, TDZ or BAP (0.1 mg/L) + 2,4-D (0.1 mg/L) produced the highest percentage of explants forming embryos (83.3%) and the highest number of embryos per treatment (31.0%).

Explants in media containing TDZ alone and under light had low callus induction frequency (10–43.3%), which increased with increasing TDZ concentration (0.1–1 mg/L). The callus formed under these treatments did not proliferate and was classified as type IV brown dead calli. The combined treatments with cytokinins and auxin resulted in high callus formation in the explant cut surfaces. Leaf tissue cultured in BAP + 2,4-D showed lower percentages of explant forming callus (63-96%) in comparison with leaf tissue cultured in TDZ + 2,4-D (96–100%). Additionally, callus morphology was significantly influenced by the light regimen (Fig. 1b–d). Both light regimens gave rise to friable soft white calli (type I) identified as embryogenic calli. Explants under dark condition formed creamy yellow nodular calli defined as type II while explants under light conditions formed nodular green calli (type III).

The presence of shoots was also as observed, with no significant statistical difference between the different treatments, although a high concentration of BAP or TDZ tended to induce hyperhydricity of the shoots. This tendency requires further studies to confirm if this can be used for faster shoot induction.

### Rooting

**Fig. 2:**
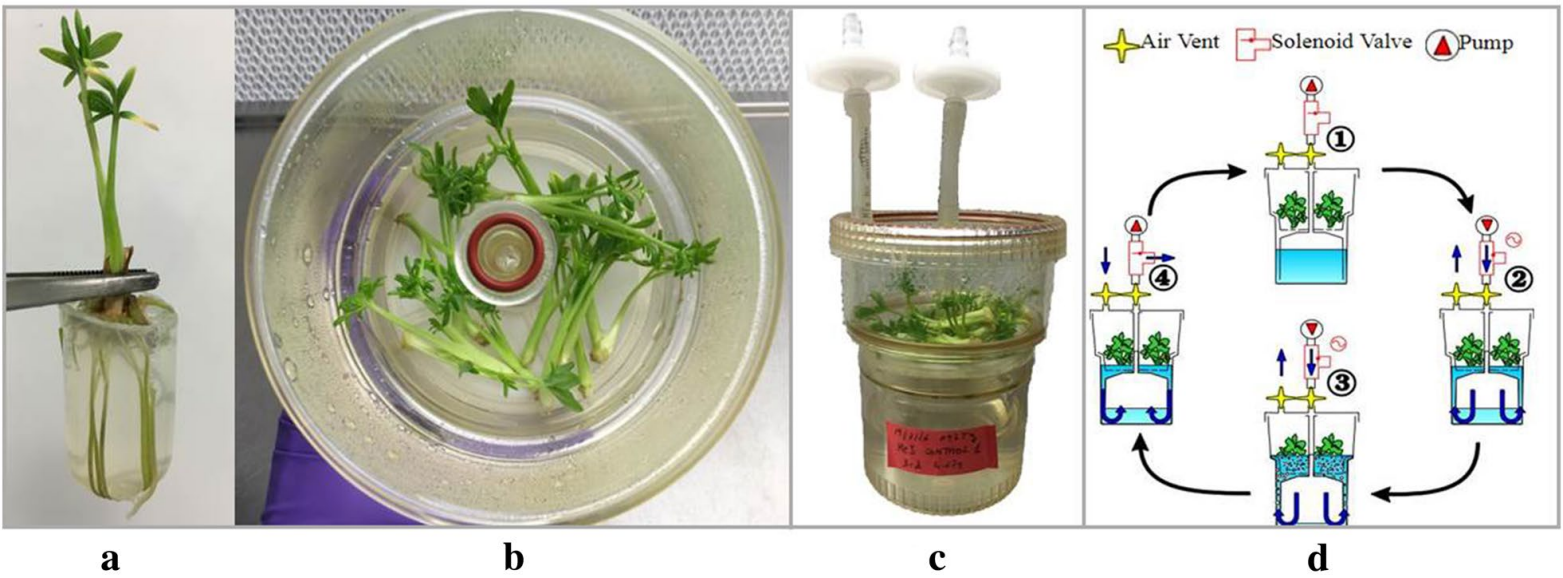
**a** *T. garganica* in vitro plant after root induction treatment (½MS + IAA (4 mg/L)) from a culture tube. **b**, **c** *T. garganica* in vitro shoots in TIB (temporary immersion bioreactor). **d** RITA^®^ operation cycle: (1) Plants are placed on a fixed raft support in the upper compartment, and the culture medium is in the lower compartment (200 mL). (2) An overpressure of 30 kPa of sterile air is applied in the lower compartment, which pushes the medium into the upper compartment, immersing the explants. (3) During 3 min every 6 h, a sterile airflow continuously agitates and oxygenates the medium allowing an inner atmosphere renewal. (4) The airflow is stopped and the culture medium drains into the lower compartment by gravity

Rooting occurred in all tested treatments, reaching 49–71% of rooted shoots after 6 weeks (Fig. 2a) (Table 2). The number of roots increased with increased concentrations of auxin. However, in treatments with the highest auxin concentration, the shoot systems were greatly damaged or died due to stress. Therefore, treatments with the lowest auxin concentrations were more suitable for rooting.

**Table 2:** In vitro rooting of *T. garganica* shoots on ½MS medium supplemented with different concentration of IAA, NAA and IBA after 6 weeks

Treatment, plant growth regulators (mg/L)			Rooted shoots (%)	Number of roots per rooted shoot	Thapsigargin (mg/g dry weight)		Nortrilobolide (mg/g dry weight)	
IAA	NAA	IBA			Shoots	Roots	Shoots	Roots
1			49^a^	4.09 ± 0.81^c^	3.56 ± 0.33^ab^	0.77 ± 0.22^a^	13.54 ± 0.64^a^	8.27 ± 0.69^a^
2			56^a^	4.80 ± 0.87^bc^	3.32 ± 0.35^abc^	0.42 ± 0.08^ab^	11.57 ± 1.18^a^	6.22 ± 0.67^a^
4			64^a^	7.21 ± 0.87^abc^	3.21 ± 0.41^abc^	0.50 ± 0.07^ab^	9.27 ± 0.94^a^	7.63 ± 0.62^a^
	1		53^a^	8.04 ± 1.14^abc^	2.24 ± 0.25^bcd^	0.38 ± 0.05^bc^	11.46 ± 0.99^a^	7.19 ± 0.84^a^
	2		51^a^	11.04 ± 2.23^ab^	1.72 ± 0.32^d^	0.41 ± 0.05^ab^	9.24 ± 1.37^a^	8.41 ± 0.29^a^
	4		49^a^	12.05 ± 2.08^a^	2.08 ± 0.21^cd^	0.30 ± 0.05^bc^	11.45 ± 0.49^a^	7.37 ± 0.66^a^
		1	60^a^	6.07 ± 1.19^abc^	3.00 ± 0.22^abcd^	0.66 ± 0.12^ab^	11.51 ± 1.41^a^	8.70 ± 0.78^a^
		2	62^a^	9.75 ± 1. 81^abc^	3.15 ± 0.21^abc^	0.48 ± 0.04^ab^	10.07 ± 0.87^a^	7.80 ± 0.62^a^
		4	71^a^	8.21 ± 1.56^abc^	3.04 ± 0.24^abcd^	0.42 ± 0.07^ab^	11.89 ± 1.14^a^	7.87 ± 0.95^a^
Half strength MS			0^b^	0^d^	3.77 ± 0.36^a^	–	13.08 ± 0.92^a^	–
MS + 1.5 BAP + 0.5 NAA			0^b^	0^d^	2.20 ± 0.11^cd^	–	10.35 ± 0.53^a^	–

After 8 weeks of rooting treatment, the samples were split into shoots and roots to quantify the amount of thapsigargin and nortrilobolide. Values represent mean ± SE. Different letters within a column indicate significant differences revealed after an ANOVA analysis followed by a Tukey’s multiple comparison test (*p* ≤ 0.05). n = 45 per treatment in number of roots; n = 6–9 for thapsigargin and nortrilobolide quantification

### Production of thapsigargins in the rooting experiment

A high-performance liquid chromatography (HPLC) analysis of extracts of the plant cultures showed different concentrations of thapsigargin depending on the treatment and tissue type (Table 2). The highest amounts of thapsigargin (3.77 mg/g DW (dry weight)) and nortrilobolide (13.08 mg/g DW) were obtained in shoots cultivated in ½MS medium without plant growth regulators. The level of thapsigargin was significant for this medium, whereas for nortrilobolide it was not. This treatment did not provide any rooting since there were no plant growth regulators. This initiated the work in TIBs, where rooting is not a prerequisite.

### Production of thapsigargins in TIBs

**Fig. 3:**
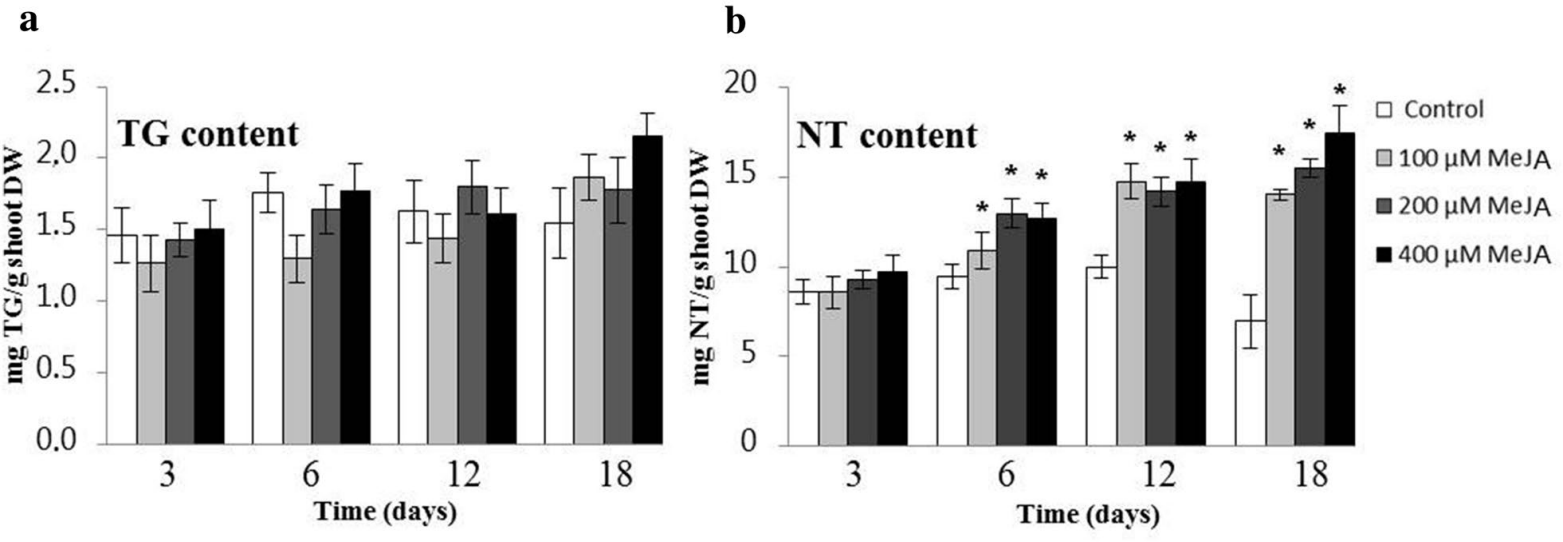
The effect of MeJA elicitation on thapsigargin and nortrilobolide content (mg/g DW) in *T. garganica* in vitro shoots in TIBs. The medium was MS salts and vitamins supplemented with 1.5 mg/L BAP and 0.5 mg/L NAA and different concentrations of MeJA (100, 200 and 400 µM). An asterisk (*) indicates a significant difference between the sample and the control. This is based on an ANOVA test followed by a Tukey’s multiple comparison test (*p* ≤ 0.05). n = 6–9 samples per treatment.TG = thapsigargin shown in (**a**), NT = nortrilobolide shown in (**b**), DW = dry weight

*Thapsia garganica* in vitro shoots were grown in TIBs for the mass production of thapsigargins (Fig. 2b). The shoot cultures were grown with the following elicitors: cellulase, alginate, yeast extract and MeJA; and the inhibitor miconazole (data not shown). Only the MeJA elicitor stimulated the production of thapsigargins (Fig. 3). Thapsigargin and notrilobolide contents were quantified by HPLC. An increase in thapsigargin and nortrilobolide was observed throughout the growth period and increased with increasing MeJA concentration. After 18 days of growth with 400 μM MeJA treatments, 2.15 mg/g DW of thapsigargin and 17.42 mg/g DW of nortrilobolide were obtained. Only the increase in nortrilobolide concentration was statistically significant (*p* < 0.0; F-value = 16.2).

**Fig. 4:**
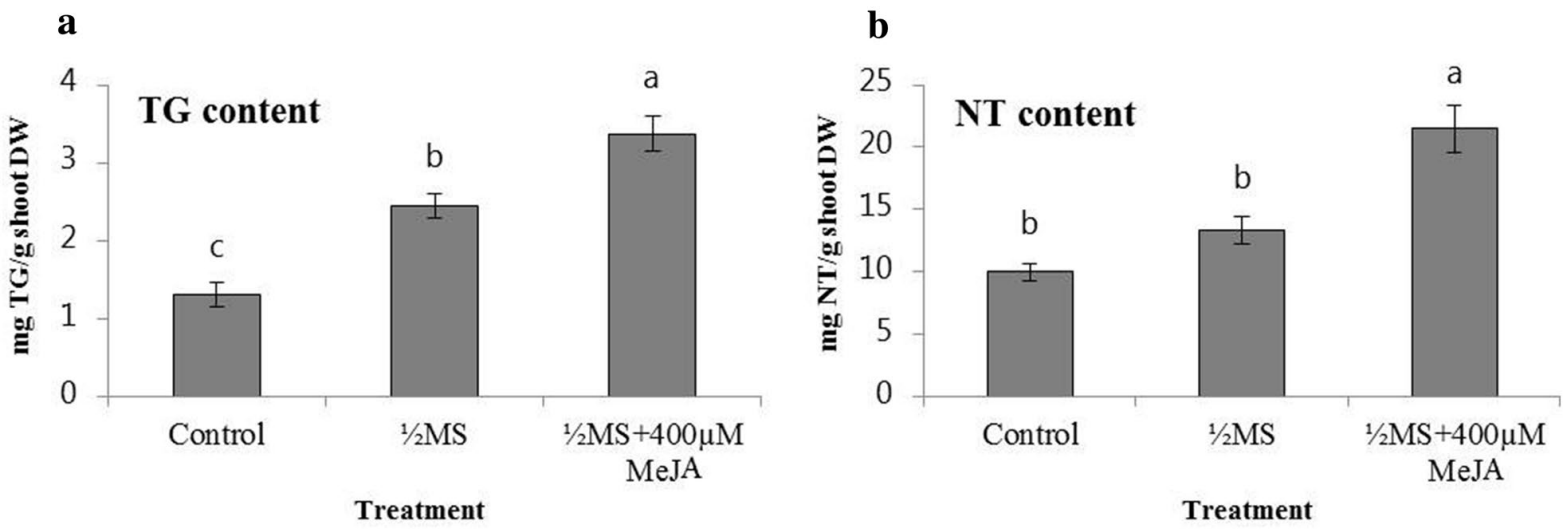
The effect of reduced nutrient supply; either alone or in combination with MeJA elicitation, on thapsigargin and nortrilobolide content (mg/g DW) in *T. garganica* in vitro shoots grown 18 days in TIBs. As controls TIBs with MS medium supplemented with BAP (1.5 mg/L) and NAA (0.5 mg/L) were used. Different letters on the bars indicate significant differences based on an ANOVA test followed by a Tukey’s multiple comparison test (*p* ≤ 0.05). n = 9 bioreactors per treatment. TG = thapsigargin shown in (**a**), NT = nortrilobolide shown in (**b**), DW = dry weight

Another study was performed with MeJA elicitation combined with reduced nutrient supply (½MS) (Fig. 4). With this treatment, the amount of thapsigargin and nortrilobolide reached 3.37 ± 0.23 mg/g DW and 21.50 ± 1.87 mg/g DW. This represents a 2.6 and 2.1 fold increase (*p* < 0.0; F-values = 32.6 and 21.06 respectively) and a total of 2.49% of the dry weight constitute these two molecules alone. For this experiment, we also observed that during the 18 days the TIBs biomass increased 3.4 fold for the control, 1.6 fold for the ½MS and 1.5 fold for ½MS + MeJA. The natural content of thapsigargins has been reported up to 0.5% in the stem and leaf. Thus, 2.49% (DW) is a very promising result.

### Identification of thapsigargins

**Fig. 5:**
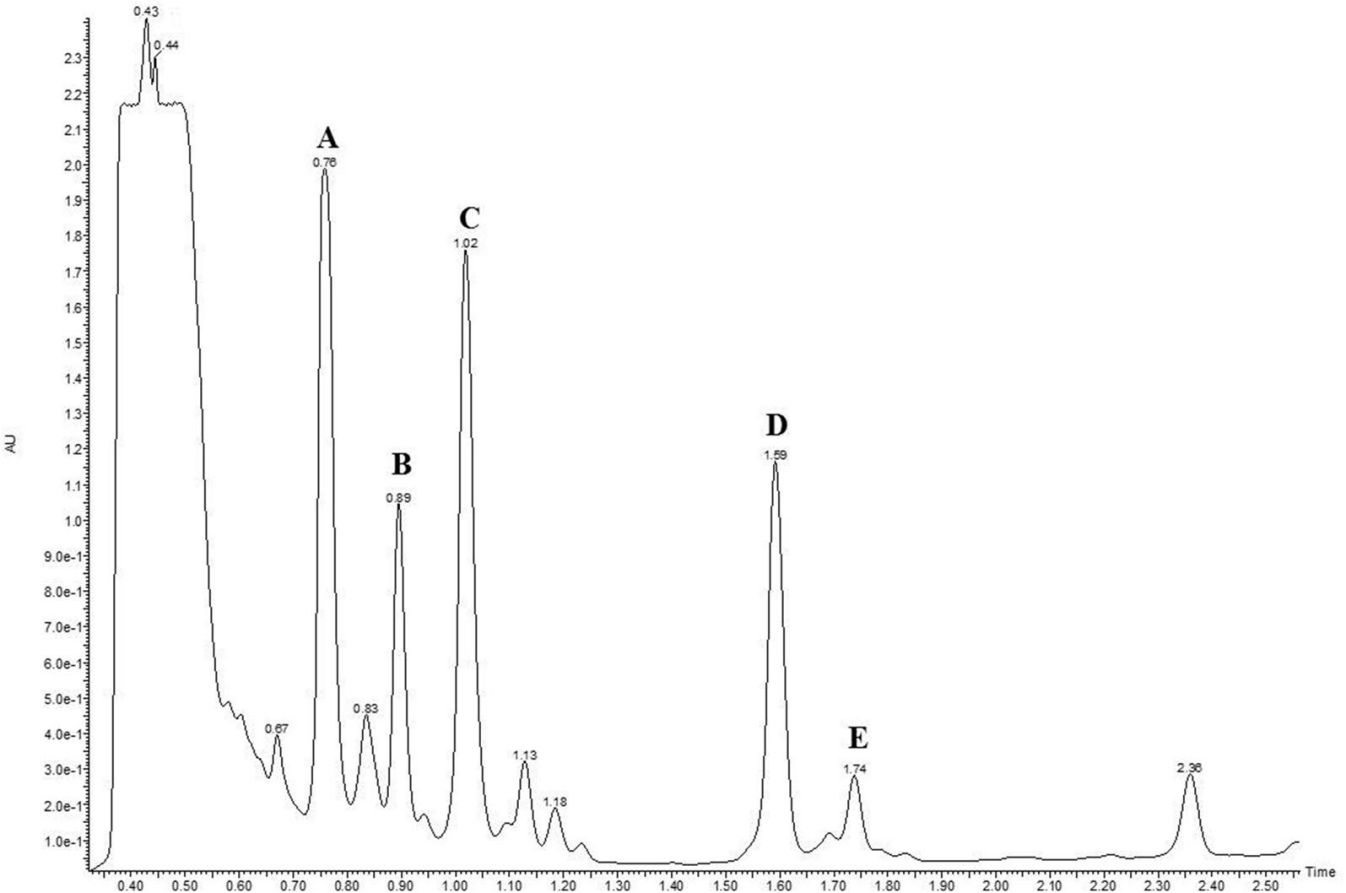
UV absorption chromatogram from a UPLC analysis of extracts of *T. garganica* in vitro plants grown in TIBs. The chromatogram shows the signal at 230 nm. (*a*) nortrilobolide, (*b*) thapsivillosin I, (*d*) thapsigargin and (*e*) thapsivillosin (*c*) Peak C could not be identified

**Fig. 6:**
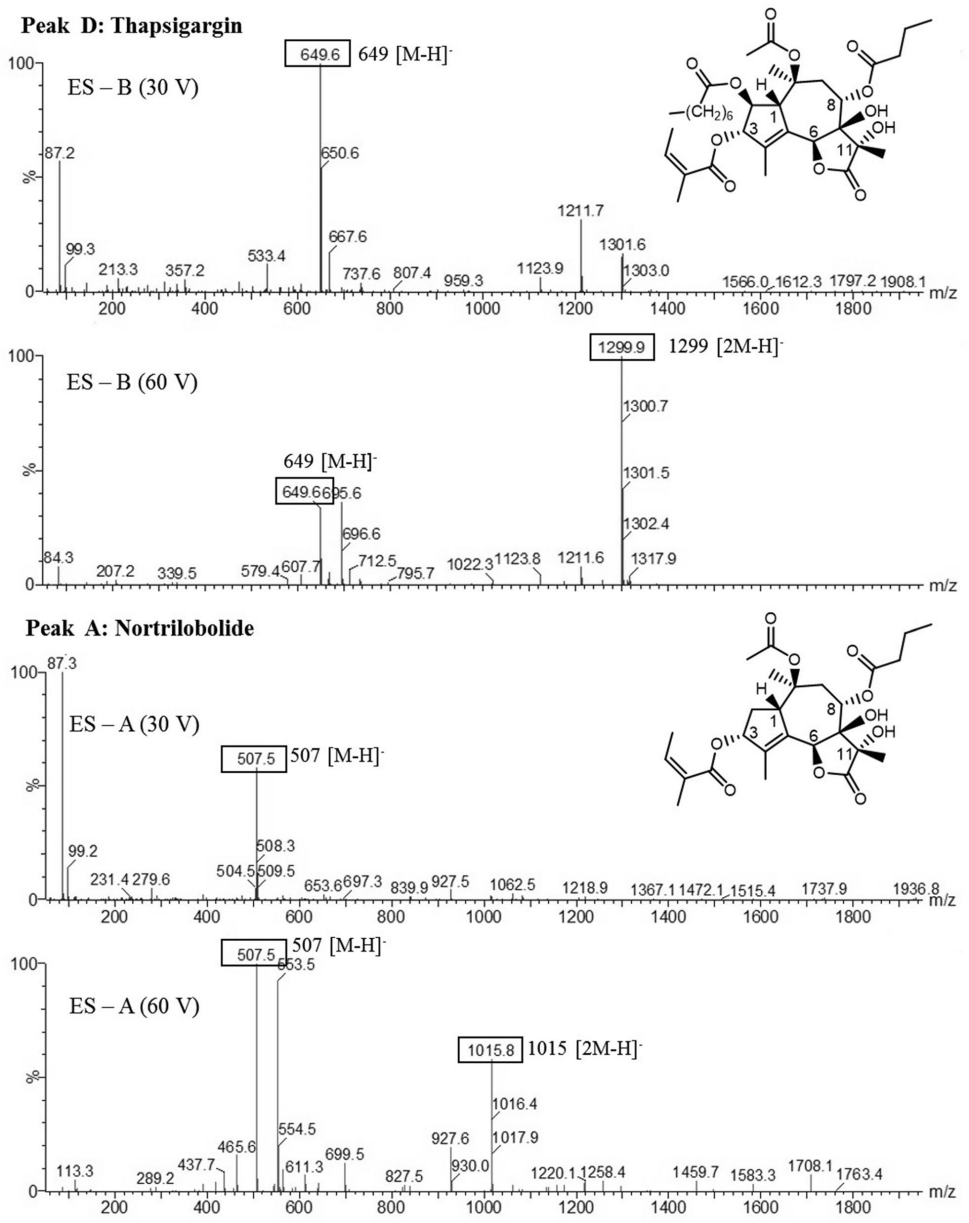
Mass spectra from the analysis of thapsigargins by UPLC analysis of extracts of *T. garganica* in vitro plants grown in TIBs. Peak A: nortrilobolide and peak D: thapsigargin in negative mode, including the chemical structure of the two compounds

In order to identify the major thapsigargins, a UPLC-MS analysis was performed. Five major peaks were separated and identified through UPLC-MS (Figs. 5, 6) and the mass spectra were compared with previously published data [18–21]. The mass spectrum of peak A corresponds to nortrilobolide (MW = 508.5) (NCBI, PubChem CID = 10097774), peak B corresponds to thapsivillosin I (MW = 605.4) (NCBI, PubChem CID = 102157434), peak D corresponds to thapsigargin (MW = 650.7) (NCBI, PubChem CID = 446378) and the MS spectrum peak E correspond to thapsivillosin C (MW = 663.5). The spectrum of peak C could not be identified. These results confirmed the presence of these molecules in in vitro cultures of *T*. *garganica*.

### Gene expression

**Fig. 7:**
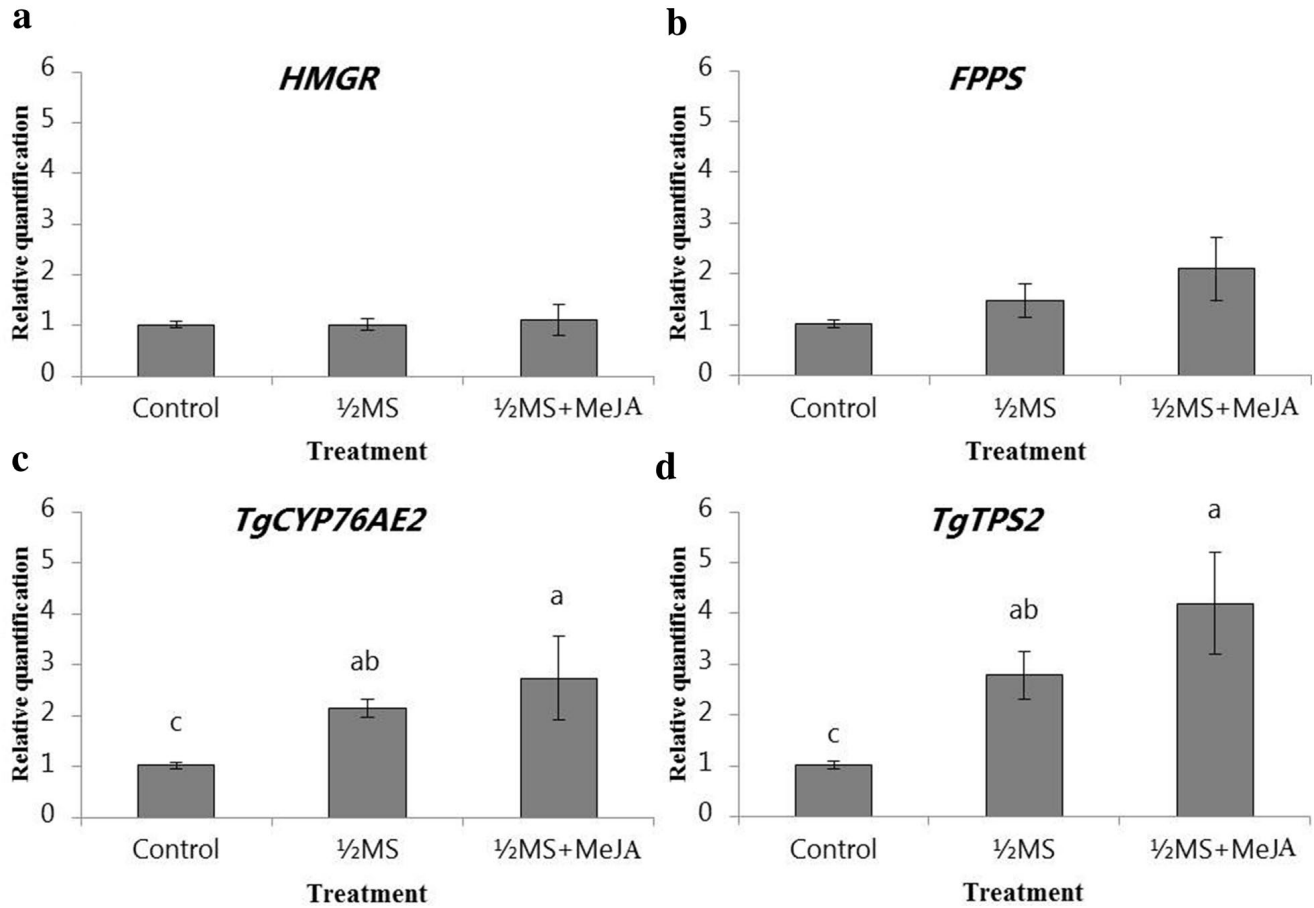
Relative quantification of *HMGR* (**a**), *FPPS* (**b**), *TgTPS2* (**d**) and *TgCYP7AE2* (**c**) expression (qRT-PCR) with three internal controls (actin, tubulin and ef1α) in *T. garganica* in vitro shoots grown in TIBs. The cultivation was performed in ½MS and ½MS + 400 µM MeJA and the RNA was extracted after 18 days of growth in TIBs. As controls TIBs with MS medium supplemented with BAP (1.5 mg/L) and NAA (0.5 mg/L) were used. The different letters on the bars indicate significant differences based on an ANOVA test followed by a Tukey’s multiple comparison test (*p* ≤ 0.05). n = 9 bioreactors per treatment

RNA transcription levels varied among treatments for the genes *TgCYP76AE2* (*p* < 0.05; F-value = 4.3) and *TgTPS2* (*p* < 0.01; F-value = 7.5) (Fig. 7c, d). The *TgCYP76AE2* gene expression increased 1.1 fold in ½MS and 1.7 fold in ½MS + 400 µM MeJ treatments, whereas the *TgTPS2* gene expression increased 1.8 and 3.2 fold, respectively. *HMGR* and *FPPS* expression levels did not significantly change with treatments, which shows a tight regulation of these two genes (Fig. 7a, b).

### Histological analysis of explants

**Fig. 8:**
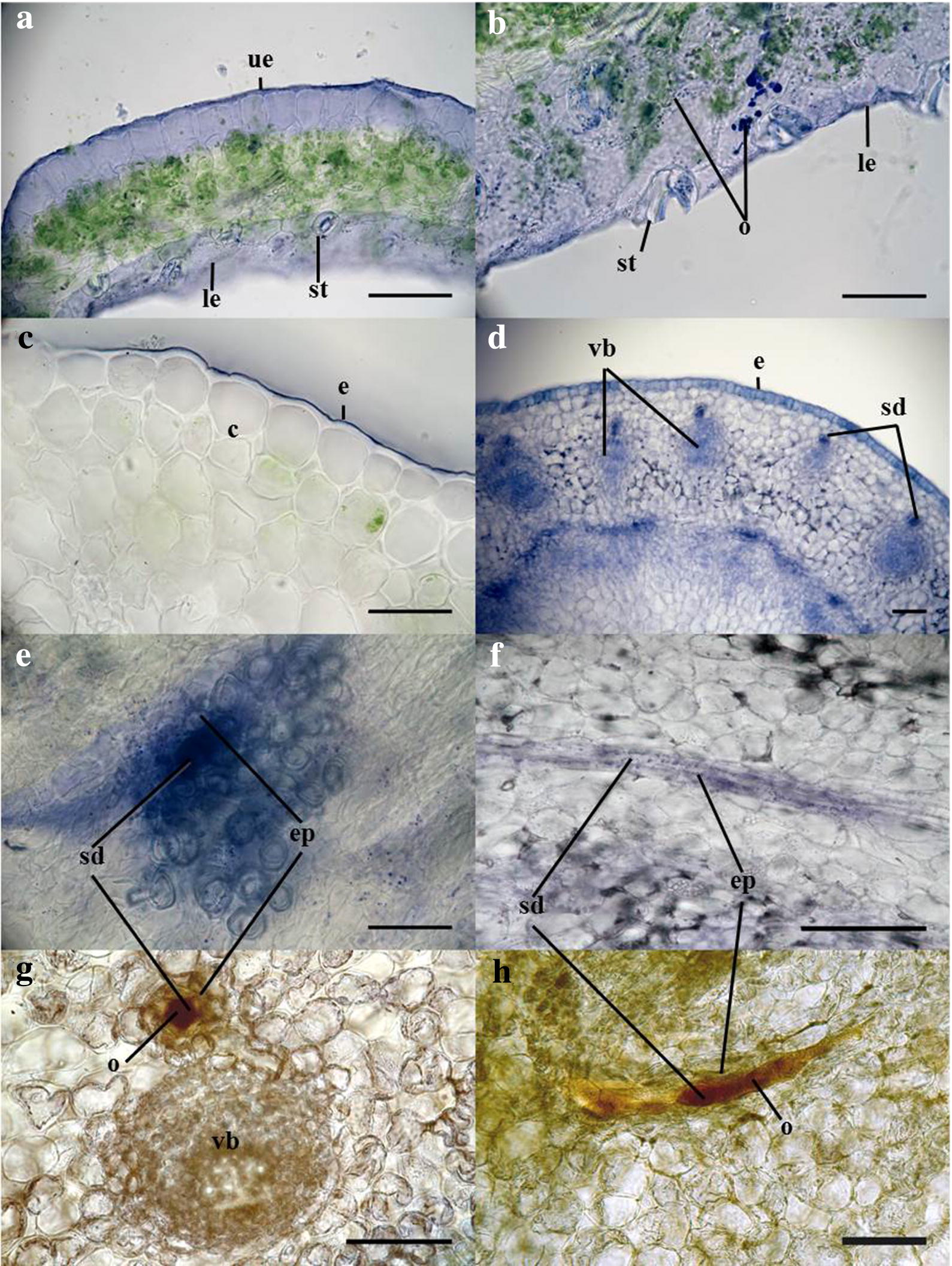
**a**–**f** NADI staining of *T. garganica* leaf and stem sections. Blue color indicates the presence of oxygenated or lipophilic compounds (e.g. terpenoids), concentrated mainly in epithelial cells, secretory ducts and in the epidermis. **a, b** Leaf cross-sections with staining visible in the epidermis. **c** upper-stem cross-section with the epidermis stained. **d** cross-section of the stem, just above callus tissue with staining concentrated in secretory ducts. **e** cross-sections of callus tissue at the base of the stems. Staining is visible in epithelial cells and secretory ducts. **f** transverse section of callus tissue showing staining of the epithelial cells along a secretory duct. **g, h** Unstained cross-section (g) and transverse section (h) of the stem to illustrate un-ruptured secretory ducts filled with resin/oil bodies *c* cortex, *e* epidermis (*le* lower epidermis, *ue* upper epidermis), *ep* epithelial cells, *o* oil bodies, *sd* secretory ducts, *st* stomata, *vb* vascular bundle. Scale bars represent 100 μm

Secretory ducts were observed to occur in the mid-rib of leaf cross-sections. The stem cross-sections revealed that the stems are surrounded by nested leaf sheaths, containing a large number of secretory ducts (Fig. 8d). The secretory ducts are surrounded by epithelial cells and radiate out from the pith of the stems to the surrounding leaf sheaths in concentric circles. The Nadi staining (Fig. 8) indicates that the epithelial cells and the secretory ducts contain terpenoids (Fig. 8e), shown by the blue color. Positive staining was also observed in the epidermis of leaves (Fig. 8a, b) and stems (Fig. 8c, d).

## Discussion

The joint action of auxins and cytokinins plays a critical role in the activation of somatic cells and cell division. This has been observed previously in *T. garganica* and other species belonging to the Apiaceae family [22–26]. Therefore, our results confirm that the cytokinin and 2,4-D combination is fundamental for *T. garganica* regeneration via somatic cells. Additionally, low concentrations of plant growth regulators gave rise to the highest numbers for somatic embryogenesis. Similar results have been reported for *Ferula assa*-*foetida* L. (also an Apiaceae), where direct and indirect embryogenesis occurred in low concentrations of 2,4-D (0.5 mg/L) and kinetin (0.2 mg/L) [25]. According to George et al., in most plant species, a low concentration of cytokinin stimulates the initiation of embryogenic cultures. In contrast, a low concentration of auxin triggers the division of the pro-embryogenic cells and their development into embryos [27]. Other studies on Apiaceae species have found that embryogenic cultures in darkness are more competent than if grown under a photoperiod [23, 28]. In the only report related to somatic embryogenesis for *T. garganica,* the authors observed that four-year-old suspension cultures spontaneously induced embryos both in darkness and in light without specifying which condition was better [14].

In our explants, treatment with TDZ led to scarce callus formation. TDZ has cytokinin-like activity and can stimulate de novo synthesis of auxin by increasing the levels of 2-(1H-indol-3-yl) acetic acid (IAA) and its precursors [29]. In *Fragaria* leaf explants, treatment with TDZ led to scarce callus formation and proliferation, and shoots became necrotic or died after 60 days [29]. Thus, the suggested de novo synthesis of auxin is not sufficient to obtain the activation. Our observations confirm that auxin and TDZ should be combined in order to obtain viable cultures.

Previous work on *T. garganica* root induction has shown a low percentage of rooted plantlets (32%) in plant growth regulator-free medium [15] and no roots when treated with auxins [16]. Later, 60% rooting frequency was obtained by treatment of 10 mg/L 1H-indole-3-butanoic acid (IBA) for 3 days prior to transfer to plant growth regulators-free medium [17]. Here we observe that 49–71% of the *T. garganica* shoots established roots in ½MS media in all auxin concentrations. The positive effect of ½MS on in vitro root induction has been previously reported for various Apiaceae species [17, 30–36]. It is known that water or nutrient deficiencies act as powerful stimulants for the activation of cell division in root meristem, in order to capture more nutrients [37]. In addition, low concentrations of nutrients provide a low osmotic pressure and ease the adaptation of the plants during acclimation. The natural auxins IAA and IBA, and the synthetic auxin 2-(1-naphthyl)acetic acid (NAA) are the most used plant growth regulators for root induction, with IBA being the most efficient auxin for Apiaceae species [33, 34, 38, 39]. However, for *T. garganica*, NAA induced the highest number of roots per rooted shoot (12.05 ± 2.08) as has also been observed for *Hydrocotyle conferta* (Apiaceae) [31]. HPLC analyses confirmed that 1 mg/L IAA was the best auxin for thapsigargin production in both shoot and root systems, but high concentrations of auxins damaged shoot systems. *T. garganica* shoot culture controls devoid of auxins did not show root induction. Surprisingly, these shoot cultures reached the highest thapsigargin levels, even more than shoots growing in the “shoot multiplication” media. Hence, a reduced nutrient supply by ½MS was used in the following experiments.

Reduced nutrient supply, via ½MS media in in vitro shoots in TIBs, yielded a 1.9 fold increase of thapsigargin content compared to full MS media (Fig. 4a). As a response to nutrient stress, plants often produce higher amounts of specialized metabolites and growth is retarded, to increase the survival rate of the individual [40]. As observed here, nutrient stress has been shown to significantly increase levels of the sesquiterpene lactone artemisinin in *Artemisia* cells whilst causing a reduction in plant biomass [41].

Elicitors have been used to either increase the production or to induce de novo synthesis of secondary metabolites in plants [42]. MeJA is a plant-specific signalling molecule synthesized in response to pathogen attack and wounding, and has been shown to be a powerful elicitor within Apiaceae species [43–49]. MeJA stimulates a stress response that often leads to the biosynthesis of specialized metabolites [50], such as terpenoids. The application of MeJA to *T. garganica* shoot cultures grown in TIBs significantly increased nortrilobolide biosynthesis, leading to a 2.5 fold increase after 18 days of treatments with 400 µM MeJA (Fig. 3b). Similar results were obtained for root cultures of *Hyoscyamus muticus* L., where the sesquiterpene production was enhanced by MeJA [51].

The joint treatment with ½MS and MeJA yielded very high concentrations of thapsigargin and nortrilobolide in *T. garganica* shoots grown in TIBs, a very promising result despite the reduced biomass (Fig. 4). The combination of the treatment and the TIB cultivation can provide a sustainable and viable production of thapsigargin and nortrilobolide. Additionally, nortrilobolide can easily be converted into thapsigargin or other relevant drug precursors by a 3-step chemical synthesis [52].

In plants, sesquiterpenoids are mainly biosynthesized by the mevalonate pathway, where HMGR and FPPS are considered key enzymes in the regulation [53]. The first specific step in the biosynthesis of sesquiterpenoids is catalyzed by sesquiterpene synthases followed by modifications in the backbone skeleton by cytochromes P450 [54]. Two sesquiterpene synthases [11] and one cytochrome P450 have been described from the root transcriptome of *T. garganica* [12]. The sesquiterpene synthase TgTPS2 is the most interesting one, with epikunzeaol being its major product [11] followed by the cytochrome TgCYP76AE2, which converts epikunzeaol to epidihydrocostunolide a likely precursor of thapsigargins [12]. We observed that the amount of thapsigargin and nortrilobolide in *T. garganica* in vitro shoots exhibited a positive and significant correlation with the expression of *TgCYP76AE2* and *TgTPS2* genes. Although the levels of expression of *TgHMGR* and *TgFPPS* did not change (Fig. 7a,b), the increase in the expression of *TgCYP76AE2* and *TgTPS2* genes (Fig. 7c,d) and the levels of sesquiterpenoids correlate with what was observed in *Arabidopsis thaliana.* Here the sesquiterpene synthases TPS21 and TPS11 were induced by jasmonate, leading to an increased emission of sesquiterpenes [55]. This shows that the levels of expression of terpene synthases and cytochromes P450 part of terpenoid biosynthesis in *Thapsia* can be induced by stress factors, and our data supports that TgTPS2 and TgCYP76AE2 are likely to be involved in thapsigargin biosynthesis.

The histochemical analysis with Nadi staining enabled the identification of secretory ducts within the stem, leaves and callus tissue of the in vitro plant material, whereas previous work focused on only root sections of *T. garganica* [12]. As previously reported [12], these secretory ducts were surrounded by epithelial cells and the Nadi staining, together with the HPLC results, provided a good indication for the presence of terpenoids and hence thapsigargins in these specialised structures. Resin exudation is known to be regulated by the epithelial cells, being used by plants to store specialised metabolites to prevent auto-toxicity [56]. The relatively large number of secretory ducts observed in the stems and callus tissue of the explants could explain the high quantity of thapsigargin measured in the HPLC analyses. It remains to be shown whether the number of ducts can be correlated with the stress factors and the expression of the terpenoid related genes, as the current data did not allow for this conclusion. Likewise, our data indicate that an induction of duct formation might be a strategy towards higher production of specialized metabolites in plants.

## Conclusions

We have shown that *T. garganica* in vitro tissue culture is an efficient production platform of thapsigargin and nortrilobolide. The in vitro culture can be formed from leaf explants. The overall setup can supply thapsigargin for commercial and pharmacological needs at industrial levels. Further process engineering is needed to optimize *T. garganica* in vitro shoot biomass production and thapsigargin levels. Our technology and method can protect this medicinal plant from future over-harvesting, as well as establishing the prerequisite cultures for plant transformation. Finally, we have confirmed that higher expression levels of *TgTPS2* and *TgCYP76AE2* genes lead to higher thapsigargin content, thus supporting their likely involvement in thapsigargin biosynthesis.

## Methods

### In vitro culture establishment

*Thapsia garganica* L. plant material was obtained from a two-year-old stock plant growing under greenhouse conditions (University of Copenhagen, Denmark). Leaves were collected to be used as explants, washed with a detergent solution and rinsed under running tap water to remove loose dirt. For surface sterilization, the explants were dipped in a 0.5% sodium hypochlorite solution with tween 20^®^ for 15 min, followed by three rinses with sterile distilled water. Leaf segments were aseptically inoculated, abaxial-side down, on Petri dishes containing 25 ml of Murashige and Skoog (MS) salts and vitamins (Duchefa Biochemie, The Netherlands), 30 g/L sucrose, 0.25 g/L phytagel and supplemented with three different plant growth regulators, N-(Phenylmethyl)-7H-purin-6-amine (BAP) or 1-phenyl-3-(1,2,3-thiadiazol-5-yl) urea (TDZ) (0.1, 0.5 or 1 mg/L), either alone or in combination with 2,4-Dichlorophenoxyacetic acid (2,4-D) (0.1 mg/L). Cultures were incubated under a 16 h light photoperiod or 24 h darkness. Each treatment consisted of 15 explants spread over three Petri dishes and the experiment was repeated twice (n = 30). Shoot organogenesis and somatic embryogenesis were investigated after culturing for a total of 12 weeks. The induction of shoots, callus, and somatic embryos was observed monthly (Table 1). The different treatments gave rise to different callus types. Type I: friable, soft white calli; type II: nodular green organogenetic and compact calli; type III: creamy, yellow nodular calli; type IV: brown dead calli. Furthermore, the amount of calli was noted from low to high (−, +, ++, +++).

### Culture media and incubation conditions

For all culture media, the pH was adjusted to 5.8 before autoclaving at 121 °C and 103 kPa for 20 min.

#### Culture establishment media

The medium was MS [57] salts and vitamins (Duchefa Biochemie, The Netherlands), 30 g/L sucrose, 0.25 g/L phytagel (Duchefa Biochemie, The Netherlands). This was supplemented with three different plant growth regulators (plant growth regulators), BAP or TDZ (0.1, 0.5 or 1 mg/L), either alone or in combination with 2,4-D (0.1 mg/L). TDZ was dissolved in NaOH and filter-sterilized through a 0.24 µM filter and added to autoclaved medium after it had cooled while the rest of plant growth regulators were added prior to autoclaving. Cultures were incubated in Petri dishes at 23 ± 2 °C under a 16 h light photoperiod (15 μmol m^−2^ s^−1^) or 24 h darkness.

#### Shoot multiplication medium

After three months, *T. garganica* shoots, buds and embryos regenerated from the leaflet explants grown in the different culture establishment media were transferred to a shoot multiplication medium defined as MS salts and vitamins, 30 g/L sucrose and 7 g/L plant agar (Duchefa Biochemie, The Netherlands). This was supplemented with 1.5 mg/L BAP and 0.5 mg/L 2-(1-naphthyl)acetic acid (NAA). Shoot cultures were multiplied in glass culture vessels, incubated at 23 ± 2 °C under a 16 h light photoperiod (15 μmol m^−2^s^−1^).

#### Rooting media

The medium was half strength MS salts (½MS) with MS vitamins, 30 g/L sucrose and 7 g/L plant agar. This medium was supplemented with 2-(1H-indol-3-yl) acetic acid (IAA), NAA and 1H-indole-3-butanoic acid (IBA) (1-4 mg/L). Cultures were incubated in 20 ml culture tubes (Fig. 2a) at 23 ± 2 °C under a 16 h light photoperiod (15 μmol m^−2^ s^−1^).

#### TIBs setup and culture media

1 Liter autoclavable RITA^®^ bioreactors were used (Fig. 2b–d) [58]. The RITA^®^ bioreactor consists of two compartments; an upper one, where the plants are placed in a raft support; and a lower one, which contains 200 mL of the culture medium. Both compartments are connected through a small tube and the raft support placed at the bottom of the upper compartment. When applying overpressure by means of the small tube, the culture medium goes up and immerses the plants in the upper compartment. During the immersion, the airflow continuously agitates and oxygenates the medium, allowing an inner atmosphere renewal. When pressure drops, the medium drains into the lower compartment by gravity. The overpressure is generated by an air pump linked to several RITA^®^ bioreactors. The air pump is controlled by a timer that regulates the frequency and the length of the overpressure; the cultures were immersed for 3 min every 6 h. The entry and exit flows were sterilized through 0.2-µm filters. The RITA^®^ bioreactors and the medium were autoclaved separately before use.

For the MeJa treatment, the medium used was MS salts and vitamins and 30 g/L sucrose. This was supplemented with 1.5 mg/L BAP and 0.5 mg/L NAA and different concentrations of MeJA (100, 200 and 400 µM). Cultures were incubated in TIBs [13] at 23 ± 2 °C under a 16 h light photoperiod (15 μmol m^−2^ s^−1^). Three bioreactors were used for each treatment, and the experiment was repeated three times (n = 9). A sample from each bioreactor was taken after 3, 6, 12 and 18 days for quantification of thapsigargin and nortrilobolide by HPLC.

For the reduced nutrient supply treatment, the medium was ½MS with MS vitamins salts and 30 g/L sucrose. This medium was free of plant growth regulators and either supplemented with 400 µM MeJA (½MS + 400 µM MeJA) or without MeJA (½MS). Cultures were incubated in TIBs [13] at 23 ± 2 °C under a 16 h light photoperiod (15 μmol m^−2^s^−1^—Photosynthetic Photon Flux Density). A sample from each bioreactor was taken after 18 days for thapsigargin and nortrilobolide HPLC quantification and gene expression analysis by qRT-PCR of the genes of interest: *HMGR*, *FPPS*, *TgTPS2* and *TgCYP76AE*.

### Shoot multiplication

*Thapsia garganica* shoots, buds and embryos regenerated from the leaflet explants were transferred to a “shoot multiplication medium” plant growth regulator to induce shoot multiplication. All regenerated shoots were left to multiply and grow in culture vessels with solid medium. Shoots cultured for approximately 3 weeks were transferred to TIBs with liquid medium, in order to use them as an efficient production platform system of thapsigargins.

### Root formation

Regenerated single shoots (4–7 cm) were transferred onto “rooting medium” (Fig. 2a). After cultivation for 4 weeks, the plants were transferred to ½MS medium free of plant growth regulators. The control was ½MS without plant growth regulators. Each treatment consisted of 15 shoots, and the experiment was repeated three times (n = 45). The percentage of root formation and the number of roots was recorded weekly over a period of 6 weeks. The content of thapsigargin and nortrilobolide was quantified by HPLC; sampling was performed after 8 weeks to allow for sufficient biomass content. The shoots and roots of the generated plants in all treatments were sampled and the quantification was performed separately in these trials (Table 2).

### Enhancement of thapsigargins production in TIBs by MeJA and reduced nutrient supply

For the treatment with MeJa, 4-7 cm shoots were added into TIBs (15 shoots per TIB) containing 200 mL of liquid MS medium supplemented with BAP (1.5 mg/L) and NAA (0.5 mg/L), and different concentrations of MeJA (100, 200 and 400 µM) (Fig. 2b). TIBs with MS medium supplemented with BAP (1.5 mg/L) and NAA (0.5 mg/L) were used to grow controls. Three bioreactors were used for each treatment, and the experiment was repeated three times (n = 9). A sample from each bioreactor was taken after 3, 6, 12 and 18 days for thapsigargin and nortrilobolide HPLC quantification.

In the reduced nutrient supply treatment, 4-7 cm shoots were added into TIBs (15 shoots per TIB) containing 200 mL of liquid ½MS medium with 400 µM MeJA or without MeJA (½MS) (Fig. 2b). Controls were cultured in MS medium supplemented with BAP (1.5 mg/L) and NAA (0.5 mg/L). Three bioreactors were used for each treatment, and the experiment was repeated three times (n = 9). A sample from each bioreactor was taken after 18 days for thapsigargin and nortrilobolide HPLC quantification and gene expression analysis by qRT-PCR of genes of interest: *HMGR, FPPS, TgTPS2 and TgCYP76AE*.

### Quantification of thapsigargin

#### Extraction of thapsigargin from plant material

Plant material from in vitro cultures (shoots and roots) was frozen at − 80 °C and dried 48 h in a freeze drier (LABCONCO FreeZone 2.5 plus). 50 mg of each dried sample was ground and extracted overnight with 1.5 mL 70% ethanol. The extract was centrifuged at 13,000 rpm for 10 min. 1 mL of the supernatant underwent evaporation by speed vacuum until dry and the residue was dissolved in 250 μL methanol 80% (concentration × 4). The extracts were then filtered through a 0.45 μm filter, placed into 1.5 mL vials and kept at − 20 °C until HPLC–DAD analysis.

Standard solutions of thapsigargin and nortrilobolide were prepared in 80% methanol in the range 12–1200 mg/L and 11–1007 mg/L respectively, corresponding to the concentration range of the plant extracts. Calibration curves were generated by integrating the peak area (230 nm) versus the concentration of standard thapsigargins. For both, the calibration curves were linear with a correlation coefficient of 0.99. The results were converted to mg of compound per g of dry weight plant (mg/g DW). The standards used in all chromatography experiments of thapsigargin and nortrilobolide and the mass spectrums of all thapsigargins were provided by Prof. Søren B. Christensen, University of Copenhagen, Denmark.

#### High-performance liquid chromatography quantification of thapsigargins

The HPLC–DAD system consisted of a quaternary pump (JASCO-2089 Plus pump), a thermoregulated autosampler set at 4 °C (Intelligent autosampler JASCO AS-2059) and a photodiode array detector (PDA) detector (UV/VIS detector JASCO MD-2018 Plus). The column temperature was 30 °C. Separation of compounds was achieved on a Luna C18 column (5 μm, 4.6 mm × 25 cm) (Phenomenex, USA). The flow rate was set to 0.5 mL/min and the mobile phase consisted of water with *o*-phosphoric acid (0.01%) as solvent A and acetonitrile with *o*-phosphoric acid (0.01%) as solvent B. The binary gradient elution was: 80–100% A (0–15 min., linear gradient), 100% A (15–25 min.) and 100–80% A (25–27.5 min., linear gradient).

#### Identification of thapsigargins by UPLC-MS

Qualitative analysis of thapsigargins from the in vitro plants was performed using a Waters Acquity TQD UPLC/MS/MS (triple quadrupole mass spectrometer) system associated with a PDA detector using the standards mentioned above. The mass spectrums was compared with those provided by Prof. Søren B. Christensen, University of Copenhagen, Denmark. The system was directed by the software MassLynx 4.1. Extracts were filtered at 0.2 µm and 7.5 μL of each extract was separated on a C18 Luna (2)-HST (100 × 2.0 mm, 2.5 μm) column (Phenomenex, USA). The flow rate was set to 0.6 mL/min and the mobile phases consisted of water with o-phosphoric acid (0.01%) for A and acetonitrile with o-phosphoric acid (0.01%) for B. The binary gradient elution was: 90% A (0–0.5 min.), 90–0% A (0.5–7 min., linear gradient), 0% A (7–10 min.), 0–95% A (10–10.2 min., linear gradient) and 95% A (10.2–13 min.). The PDA swept wavelengths from 200 to 400 nm with a 2.4 resolution. Mass spectra were obtained in positive and negative modes, using an electrospray ionization (ESI) source on a triple quadrupole instrument (Waters Acquity) in full scan (50–2000 m/z). The conditions were as follows, capillary voltage: 3000 V, cone voltages: 30 and 60 V, desolvation temperature: 450 °C, source temperature: 150 °C, gas flow cone: 50 l/min, desolvation gas: 800 l/min.

### Gene expression analysis by qRT-PCR

RNA transcription levels of genes of interest: *HMGR*, *FPPS*, *TgTPS2* and *TgCYP76AE2* were measured for *T. garganica* in vitro shoots growing in TIBs under reduced nutrient supply (½MS) or under reduced nutrient supply in combination with MeJA (½MS + 400 µM MeJA). Controls were cultured in TIBs with MS medium supplemented with BAP (1.5 mg/L) and NAA (0.5 mg/L).

#### RNA isolation and primer design

Total RNA was isolated from 75 to 85 mg samples from elicited and non-elicited shoots. RNA was extracted with the Spectrum™ Plant Total RNA kit (Sigma-Aldrich, Copenhagen, Denmark) and was treated with DNaseI Amplification Grade (Sigma-Aldrich, Copenhagen, Denmark). To determine nucleic acid purity, 260/280 nm ratios were measured using an Agilent RNA 6000 Nano kit (Agilent Technologies, Copenhagen, Denmark).

Three reference genes were selected for normalization: actin, elongation factor (ef1α), and tubulin. The previously published *T. garganica* root transcriptome data (SRX096991) was used for primer design. *Daucus carota* sequences, acquired from NCBI database, of genes: actin, tubulin, *FPP* and *HMGR* were used to do a BLAST against *T. garganica* root transcriptome, while *Solanum tuberosum* sequence was used for ef1α gene [12]. *Daucus* is the sister genus to *Thapsia* thus was an obvious choice for the design of primers. For the ef1α *Solanum* was the closest relative available. Primer design of *TgCYP76AE2* and *TgTPS2* was based on the published sequences [11, 12] (Additional file 1: Table S1).

#### Two steps qRT-PCR

A total of 500 ng of each RNA sample was reverse transcribed to cDNA with IScript™ cDNA Synthesis kit (BIO-RAD, Copenhagen, Denmark). Two-step qRT-PCR (as specified by the kit supplier) was performed using QuantiFast^®^ SYBR^®^ Green PCR (Qiagen, Copenhagen, Denmark) according to the manufacturer’s protocol. The following amplification program was used: 95 °C for 5 min, 40 cycles at 95 °C for 10 s followed by 60 °C for 30 s. Samples were amplified in duplicate from the same RNA isolation. qRT-PCRs were performed using 3 biological replicates from different bioreactors and the experiment was repeated 3 times. Therefore, 9 biological replicates (with 18 data points) spread out in three different elicitation experiments were analyzed.

qRT-PCR efficiency, E, was estimated for each gene by generating standard curves by plotting quantification cycle (C_q_) values (y) against the log of a series of cDNA dilutions (x). For this, a cDNA sample was used as template in a range of 20, 4, 2, 1 and 0.5 ng. The qRT-PCR efficiencies were calculated from the slope (a) of the linear regression equations of the standard curves, along with the regression coefficient (R^2^). The equation used was: E = 10^(−1/a)^ [59]. E values in a range of 1.90–2.10 (PCR efficiency between 90 and 110%) with a regression coefficient below 0.02 are acceptable. All PCR efficiencies displayed between 96 and 104% (Additional file 1: Table S1).

#### Determination of reference gene expression stability, data analysis and normalization using geNorm

The stability of the reference genes and normalization factors were evaluated with geNorm V.3.1 (www.genorm.cmgg.be). The geNorm algorithm is based on the pairwise variation of a single reference candidate gene relative to all other genes, assuming that the expression ratios of the two ideal reference genes are identical in all samples regardless of the conditions tested [60]. geNorm calculates two parameters: the expression stability value of reference gene (M) and the coefficient of variation of the normalized reference gene relative quantities (CV) [61]. The cut-off proposed for typical stable reference genes for M and CV values are 0.5 and 0.25 respectively, however, for more heterogeneous samples, M and CV values can increase to 1 and 0.5 respectively [61].

Normalization factors were calculated following the Δ-Δ-Ct method as previously described [62] and using a reference target normalization approach with the three reference genes: actin, elongation factor (ef1α), and tubulin [60].

#### Verification of amplified products and sequencing reactions

PCR product size was checked on a 2% agarose gel and melting curves showed a singled product for all genes. To verify sequences of amplification products, PCRs for each primer set were performed. PCR reactions comprised: 0.5 µM of primers, 0.02 U/µl of Phusion High-Fidelity DNA Polymerase (Thermo Scientific, Copenhagen, Denmark), 2 mM of dNTP mix, 1 × Phusion HF Buffer (Thermo Scientific, Copenhagen, Denmark) and 10 ng cDNA in a total volume of 20 µl. The following amplification program was used: 98 °C for 30 s and 34 cycles at 98 °C for 5 s, 58 °C for10 s and 72 °C for 20 s followed by 72 °C for 5 min. Amplified products were cloned with CloneJET PCR Cloning Kit (Thermo Scientific, Copenhagen, Denmark) for sequencing, according to the manufacturer’s instructions. *E. coli* strain (DH5α) was transformed with the ligation products, 4 colonies for each gene were analyzed by colony PCR with plasmid reverse specific primer (5′-AAGAACATCGATTTTCCATGGCAG-3′) at an annealing temperature of 60 °C. One colony per gene was selected for sequencing (Eurofins), previous plasmid preparation (GenElute Plasmid Miniprep kit, Sigma-Aldrich, Copenhagen, Denmark). Sequences of amplification products were compared through a BLAST analysis.

### Histological analysis of explants

A histological analysis was performed to investigate the tissue structure of the in vitro shoots, and a histochemical analysis with Nadi was carried out to identify secretory structures storing terpenoids. Nadi staining has been described as a tool to detect terpenoids [63] and has been used to this effect in a number of studies [64, 65]. The method is a reaction that is believed to result from the oxidation of naphthalen-1-ol and N,N-dimethylbenzene-1,4-diamine by cytochrome oxidase enzymes [66]. In the presence of monoterpenes and sesquiterpenes, the diamine is enzymatically oxidised, resulting in a free radical that combines with naphthalen-1-ol to form 4-(4-hydroxyphenyl)iminocyclohexa-2,5-dien-1-one blue [66, 67]. For the histological analysis, 65 µm sections of the stems, leaves and callus tissue were made using a vibratome (Microtome, HM 650 V vibratome (Thermo Scientific). The sections were collected in tubes containing either 0.05 M phosphate buffer (pH 7.2), to keep unstained, or in Nadi reagent (1% naphthalen-1-ol with 1% N,N-Dimethylbenzene-1,4-diamine in 0.05 M phosphate buffer, pH 7.2; immersed for approximately 30 min). The sections were then mounted on glass slides and observed under a Leica DMR HC microscope through ×5, ×10, ×20, ×40 dry objectives and a ×100 oil immersion objective. The resulting images were processed and analysed using the Fiji platform on ImageJ [68].

### Statistical analysis

All experiments were repeated 3 times, except for the regeneration from *Thapsia* leaves that was repeated twice. The data are reported as mean ± SE (standard error) including all data points. Means were subjected to a one-way analysis of variance (ANOVA) test followed by a Tukey’s multiple comparison test (*p* ≤ 0.05).

## Additional file

**Additional file 1: Table S1.** Reference and target gene: primer sequences, amplicon length, PCR efficiency and reference gene expression stability.
