# Supplementary material for: Use of a temporary immersion bioreactor system for the sustainable production of thapsigargin in shoot cultures of Thapsia garganica
Source: Plant Methods. 2018 Sep 8;14:79. doi: 10.1186/s13007-018-0346-z (PMC6128993; doi:10.1186/s13007-018-0346-z)
Supplement: Supplementary file 1 — Additional file 1: Table S1. Reference and target gene: primer sequences, amplicon length, PCR efficiency and reference gene expression stability. [file 13007_2018_346_MOESM1_ESM.docx]

**Table S1.** Reference and target gene: primer sequences, amplicon length, PCR efficiency and reference gene expression stability.

| Gene (Accession Number) | Primer sequence | Amplicon length (bp) | | Ta (°C) | Tm (°C) | PCR efficiency (E/%) | Regression Coefficient (R^2^) | M  (± SE) | CV  (± SE) |
| --- | --- | --- | --- | --- | --- | --- | --- | --- | --- |
| Reference genes | | | | | | | | | |
| Actin(X17526) | F: GGACGTACAACCGGTATTGTG | | 199 | 56 | 82.5 | 1.94/93.95 | 0.996 | 0.92±0.03 | 0.33±0.02 |
|  | R: CAATTTCCCGCTCAGCAGTG | |  |  |  |  |  |  |  |
| Tubulin(X16608) | F: GTTCAGAAGAGTGAGTGAGCAATTT | | 196 | 55.5 | 80 | 1.97/97.24 | 0.996 | 0.98±0.07 | 0.40±0.04 |
|  | R: CTCATACTCATACTCATCCTCCTCG | |  |  |  |  |  |  |  |
| ef1α(AB061263) | F: AACAGCGAACCTACCAAGGG | | 200 | 57.6 | 83 | 2.08/108.16 | 0.975 | 1.02±0.11 | 0.42±0.01 |
|  | R: GCCACACCTCTCACATTGCT | |  |  |  |  |  |  |  |
| Target genes | | | | | | | | | |
| FPPS(XP017248920) | F: AGCGCTTGAACTTTCTAATGAGG | | 199 | 56 | 78.5 | 1.97/96.56 | 0.999 | - | - |
|  | R: TGCTTGCACGTATGTGTTTGG | |  |  |  |  |  |  |  |
| HMGR(XP017253170) | F: TGCTTGCCTGAACCTACTGG | | 200 | 56.5 | 83.5 | 2.01/101.28 | 0.998 | - | - |
|  | R: GGCAGGCAATTGTGGACATA | |  |  |  |  |  |  |  |
| TgTPS2 (*) | F: TCTGGGGCTGTCTTACCACT | | 204 | 58.3 | 79 | 1.98/97.65 | 0.997 | - | - |
|  | R: TCATTGAACTTCCCGTCGCT | |  |  |  |  |  |  |  |
| TgCYP76AE2 (*) | F: ATGCTTGGGCAATCGGAAGA | | 190 | 60 | 81.5 | 2.01/100.68 | 0.997 | - | - |
|  | R: AACGTGCCAAGAAGTAGGGG | |  |  |  |  |  |  |  |
